# Impact of Mononuclear Cell Infiltration on Chondrodestructive MMP/ADAMTS Production in Osteoarthritic Knee Joints—An Ex Vivo Study

**DOI:** 10.3390/jcm9051279

**Published:** 2020-04-28

**Authors:** Hadrian Platzer, Timo A. Nees, Tobias Reiner, Elena Tripel, Simone Gantz, Sébastien Hagmann, Babak Moradi, Nils Rosshirt

**Affiliations:** Clinic for Orthopedics and Trauma Surgery, Center for Orthopedics, Trauma Surgery and Spinal Cord Injury, Heidelberg University Hospital, Schlierbacher Landstr. 200a, 69118 Heidelberg, Germany; hadrian.platzer@med.uni-heidelberg.de (H.P.); timo.nees@med.uni-heidelberg.de (T.A.N.); tobias.reiner@med.uni-heidelberg.de (T.R.); elena.tripel@med.uni-heidelberg.de (E.T.); simone.gantz@med.uni-heidelberg.de (S.G.); sebastien.hagmann@med.uni-heidelberg.de (S.H.); babak.moradi@med.uni-heidelberg.de (B.M.)

**Keywords:** osteoarthritis, T cells, macrophages, chondrocytes, synovial membrane, MMP, ADAMTS, chondrodestruction

## Abstract

Progressive loss of joint function in osteoarthritis (OA) is driven by degenerative and inflammatory processes and their complex interaction. Decoding the link between degeneration and inflammation is one of the most exciting approaches in understanding OA pathophysiology and holds the promise to open new therapeutic avenues. The overarching goal of this project was to analyze the impact of mononuclear cells (MNC) on enzymatic chondrodestructive processes (MMP/ADAMTS) in OA. Synovial membrane (SM), articular cartilage (AC) and peripheral blood (PB) were obtained from a total of 21 patients with advanced knee OA who underwent arthroplastic surgery. In supernatants of native synovial cell cultures, T cell-depleted synovial cell cultures and macrophage-depleted synovial cell cultures, the concentrations of various metalloproteinases were examined by Enzyme Linked Immunosorbent Assay (ELISA). Furthermore, ELISA was used to analyze concentrations of metalloproteinases in supernatants of chondrocyte monocultures and chondrocyte co-cultures with CD4^+^CD127^dim/-^ enriched peripheral blood mononuclear cells (PBMC), Treg depleted CD4^+^CD25^-^CD127^dim/-^ enriched PBMC and CD4^+^CD25^+^CD127^dim/-^ Treg. Compared to native synovial cell culture, T cell depletion led to significantly lower levels of MMP-1, MMP-3 and MMP-9 and macrophage depletion led to a significant decline of MMP-1, MMP-3, MMP-9 and ADAMTS-5 concentration. Compared to T cell depletion, macrophage depletion resulted in a significantly stronger reduction of MMP-1, MMP-3, MMP-9 and ADAMTS-5. In chondrocyte co-culture with CD4^+^CD127^dim/-^ enriched PBMC the concentration of MMP-1 and ADAMTS-5 was significantly increased compared to chondrocyte monoculture. No significant differences were found between chondrocyte monoculture and chondrocyte co-culture with Treg as well as between co-culture with CD4^+^CD127^dim/-^ enriched PBMC containing Treg and co-culture with Treg-depleted CD4^+^CD25^-^CD127^dim/-^ enriched PBMC. In conclusion, our data suggests that both synovial macrophages and T cells have a catabolic potential by inducing the release of chondrodestructive metalloproteinases in OA synovium. This study also supports the hypothesis that MNC affect the release of metalloproteinases by chondrocytes and are hereby involved in the cartilage-induced chondrodestructive process. In this study no suppressive effect of Treg was shown.

## 1. Introduction

Osteoarthritis (OA) is the most frequent joint disease worldwide [1] leading to severe physical and social limitations [2,3,4]. In addition, OA leads to a large economic burden due to enormous direct and indirect costs [5,6,7,8,9]. The fact that there is still no causal therapy for OA is due to our decade-long mechanical approach towards OA pathophysiology and the negligence of its underlying cellular and molecular processes. Breaking up with this one-dimensional approach, it is increasingly recognized that OA is not a mere degenerative disease. Instead, recent studies classify it as a disease of the whole joint [10], caused by a complex pathophysiology leading to an imbalance of anabolic and catabolic mechanisms. The enzymatic process is driven by Matrix-metalloproteinases (MMP) and “a disintegrin and metalloproteinase with thrombospondin motifs” (ADAMTS), which are capable of degrading the main components of cartilage, in particular collagen and aggrecan [11,12,13,14,15,16]. While chondrocytes were long regarded as metabolically inactive in the simplified “wear and tear” explanatory model, it is assumed that chondrocytes contribute to the enzymatic burden of OA joints by producing inflammatory cytokines and MMP [17]. Synovial cells, which are necessary for normal joint homeostasis by exerting phagocytosis or production of lubricants, seem to change their functional status in OA disease towards an inflammatory and chondrodestructive phenotype. Recently it was shown in a traumatically induced OA animal model that synovial production of pro-inflammatory interleukins was increased whereas production of chondroprotective agents was reduced [18]. Furthermore, synovial cells are considered to be significantly involved in MMP and ADAMTS production [11,19,20].

Accepting these enzymatic processes to be of utmost importance in OA pathophysiology, the regulatory mechanisms of MMP and ADAMTS production, release and metabolism need to be understood. It is hypothesized that synovial infiltration with mononuclear cells (MNC) contributes to the catabolic enzymatic load in OA joints through its influence on MMP [19,20]. Synovitis has been detected by magnetic resonance imaging (MRI) in about 90% of patients with symptomatic knee OA [21] and findings by a recent publication suggests that synovitis might be a predictive factor for cartilage damage [22]. Further results suggest that pronounced synovitis is an independent risk factor for the onset of knee OA [23]. Macrophages and T cells have been identified in OA synovium as the main MNCs [24,25,26,27,28] and the focus of our group in previous studies has been to characterize the infiltrating T cell subsets present in synovial fluid and membrane of OA knee joints [27,29].

Among the different T cell subtypes, regulatory CD4^+^CD25^+^CD127^low/-^ T cells (Treg) [30,31], which are known for their anti-inflammatory characteristics and their role in the maintenance of self-tolerance [32,33,34,35], were detected in OA synovium and synovial fluid [29]. Even though the characterization of the mononuclear cell infiltrate and the enzymatic profile in OA joints is proceeding, the interaction between inflammation and degradation is still a blank spot. In particular, the role of synovial T cells and whether they are actively involved in the cartilage degradation process by inducing the release of MMP and ADAMTS is completely unknown. A better understanding of inflammatory cascades—including identification of the key enzymes and cells responsible for metabolic imbalance and cartilage degradation—is an opportunity to establish new therapeutic strategies with the overarching goal of treating OA not only symptomatically but also causally.

The main objective of this ex vivo study was to analyze the influence of synovial macrophage and T cells on MMP and ADAMTS release. Considering the literature, we focused on MMP-1, MMP-3, MMP-9, MMP-13 and ADAMTS-4, ADAMTS-5 and ADAMTS-7, which were associated with catabolic turnover in OA [11,12,13,14,15,16]. The second aim of this study was to investigate catabolic chondrocyte activity in patients with advanced knee OA and how this activity depends on the presence of different subtypes of CD4^+^-enriched PBMC. In addition, we wanted to investigate the effect of Tregs (successful isolation of Treg from PBMC is shown in Supplementary Figure S1) and whether they are able to suppress catabolic activity in a co-culture experiment.

## 2. Materials and Methods

### 2.1. Study Population

The demographic parameters of the study population are shown in Table 1. Twenty-one patients with a mean age of 66 ± 12.5 years and advanced knee OA were enrolled in this study. OA was defined according to the American College of Rheumatology criteria. All included patients had K&L grade 3 or 4 and underwent surgery for unicompartmental knee arthroplasty (UKA) or total knee arthroplasty (TKA) at the University Hospital of Heidelberg. Blood examination before surgery confirmed that none of the patients had signs of systemic inflammation (CRP, Leucocytes). None of the patients reported intake of disease-modifying anti-rheumatic drugs (DMARDs) or intraarticular injections three months prior to the operation. The ethics committee of the University of Heidelberg (approval code: S-156/2014) approved this study and informed consent from all patients was obtained prior to study enrolment.

### 2.2. Sample Collection

Synovial membrane (SM) and articular cartilage (AC) of the tibial plateau were harvested at the time of surgery and transported to our laboratory under sterile conditions for further processing. Matched blood samples were taken five days post-surgery.

### 2.3. Cell Preparation and Isolation

SM samples were rinsed twice with phosphate-buffered saline (PBS), minced finely with sterilized scissors, diluted in 6.7 mL RPMI 1640 culture medium (Thermo Fisher Scientific, Waltham, MA, USA) supplemented with 10% fetal calf serum (FCS) (Biochrom, Berlin, Germany) and 1% streptomycin-penicillin (10,000 µg/mL, Biochrom, Berlin, Germany) and digested with 333 µL/g collagenase B (20 mg/mL, Roche Diagnostics, Suisse) at 37 °C for 2 h. The cell suspension was filtered through a 100-µm cell strainer (Becton Dickinson, Heidelberg, Germany) and further through a 41-µm net filter (Merck KGaA, Darmstadt, Germany) to remove any undigested tissue. Cells were washed and resuspended in a 12 mL MACS buffer. CD3^+^ (order number: 130-050-101) and CD14^+^ (order number: 130-050-201) cell depletion was performed by magnetic activated cell sorting (MACS) according to manufacturer’s instructions (Miltenyi Biotec, Bergisch Gladbach, Germany). The use of LS-columns instead of recommended LD-columns optimized the depletion process. To prevent non-specific binding, 20 µL of FCR-blocking reagent was added (Miltenyi Biotec, Bergisch Gladbach, Germany).

MNC were isolated from heparin anti-coagulate peripheral blood (PB) using Ficoll-PaqueTM PLUS (GE Healthcare, USA) density gradient centrifugation. CD4^+^CD127^dim/-^-enriched PBMC, CD4^+^CD25^+^CD127^dim/-^ regulatory T cells (Treg) and a population of Treg-depleted, CD4^+^CD25^-^CD127^dim/-^-enriched PBMC were isolated using the CD4^+^CD25^+^CD127^dim/-^ Regulatory T cell Isolation Kit II (Miltenyi Biotec, Bergisch Gladbach, Germany; order number: 130-094-775) according to the manufacturer’s instructions.

Autologous samples of tibial plateau resections were washed with PBS, cartilage was dissected from underlying bone with sterile scalpels and minced finely. Cartilage was diluted in 8 mL/g DMEM (supplemented with 10% FCS and 1% Penicillin/Streptomycin) and digested with 1 mL collagenase B (20 mg/mL) and 1ml hyaluronidase (1 mg/mL) (Sigma-Aldrich, St. Louis, MO, USA) for 12 h at 37 °C. The cell suspension was filtered through a 100-µm cell strainer and washed once. Then, 10^6^ chondrocytes were incubated in 25 mL DMEM (supplemented with 10% FCS and 1% Penicillin/Streptomycin) with 5% CO_2_ at 37 °C for five days. DMEM was replaced on the second day after surgery. On the fifth day after surgery, cells were detached using Trypsin/EDTA, washed once and diluted in RPMI (supplemented with 10% FCS and 1% Penicillin/Streptomycin) at 10^6^ cells/mL concentration.

### 2.4. Culturing of the Native Synovial Membrane and Depletion Assays

Native and CD3^+^, resp. CD14^+^-depleted SM cell suspensions were suspended in RPMI medium (supplemented with 10% FCS and 1% Penicillin/Streptomycin) and cultured in a 12-well plate at 10^5^–10^6^ cells/mL concentration at 37 °C and 5% CO2 for 24 h. Supernatants were frozen at −80 °C until further use.

### 2.5. Chondrocyte Monoculture and Co-Culture with Mononuclear Cells

Suspensions of the PBMC populations described above were washed and diluted in RPMI medium (supplemented with 10% FCS and 1% penicillin/streptomycin). Monocultures of 5 × 10^4^ chondrocytes and direct co-cultures of 5 × 10^4^ chondrocytes and 10^5^ mononuclear cells were cultured on 24-well plates at 37 °C. Supernatants were harvested after 48 h and stored at −80 °C until further use.

### 2.6. Flow Cytometry Analysis of Cell Surface Markers

Multi-color flow cytometry was performed to identify mononuclear cells by their specific surface markers in samples of both native SM assays and SM depletion assays. In brief, cells were washed with MACS staining buffer and incubated for 5 min at 4 °C with 10 µL of FCS blocking reagent to avoid non-specific binding. Cells were then incubated with R-phycoerythrin (PE) conjugated antibodies against CD45 (Clone: 5B1, Miltenyi Biotec, Bergisch Gladbach, Germany), fluorescein isothiocyanate (FITC) conjugated antibodies against CD14 (Clone: M5E2) and Allophycocyanin (APC) conjugated antibodies against CD3 (Clone: HIT3a). After 30 min incubation at 4 °C, cells were washed and resuspended in 200 μL MACS staining buffer. Immediately after the addition of 1 µL 7-aminoactinomycin D (7-AAD) to exclude cell debris and dead cells, flow cytometric analysis was performed using MACSQuant Analyzer (Miltenyi Biotec, Bergisch Gladbach, Germany). The data were analyzed using FlowJo, version 9.8.1 (FlowJo, Ashland, OR, USA). Alive cells were gated by their forward/side-scatter profile. MNC were defined by their specific surface expression profile as CD45^+^CD14^+^ macrophages and CD45^+^CD3^+^ T cells. Antibodies and cell preparation solutions were purchased from BD Biosciences, Franklin Lakes, NJ, USA, if not stated otherwise.

### 2.7. Enzyme Linked Immunosorbent Assay (ELISA)

ELISA was utilized to measure enzyme concentration of MMP-1 (Sigma, RAB0361-1KT), MMP-3 (Sigma RAB0367-1KT), MMP-9 (Sigma, RAB0372-1KT), MMP-13 (Sigma, RAB0364-1KT), ADAMTS-4 (LOXO, 6SEK204HU), ADAMTS-5 (LOXO, 6SEK205HU) and ADAMTS-7 (LOXO, 6SEB974HU) in supernatants of cell cultures following the manufacturer’s instructions. Photometric analysis was performed using Dynatech Laboratories MRX Microplate Reader (Dynex Technologies, Denkendorf, Germany).

### 2.8. Statistical Analysis

Data are presented as arithmetic mean ± standard deviation, if not stated otherwise. Gaussian distribution was assessed using Shapiro–Wilk test. Since data analysis showed not normally distributed data in a few cases of the synovial cell culture experiment, Friedman and post-hoc Wilcoxon signed-ranks tests with Bonferroni adjustment were used. For the chondrocyte culture experiment, the Shapiro–Wilk test showed no deviations from the normal distribution. Therefore a repeated measures ANOVA with Bonferroni adjustment was carried out. Statistical analysis was performed using SPSS (IBM Corp. Released 2015. IBM SPSS Statistics for Windows, Version 23.0., Armonk, NY, USA). GraphPad Prism (Version 5.01 for windows, La Jolla, California, USA) was utilized to graph the mean values and standard deviation of enzyme concentrations (pg/mL). *P*-values are marked as follows: * *p* < 0.05, ** *p* < 0.01, *** *p* < 0.001. All reported *p*-values are two-tailed.

## 3. Results

### 3.1. Synovial MMP and ADAMTS Production

**Figure 1:**
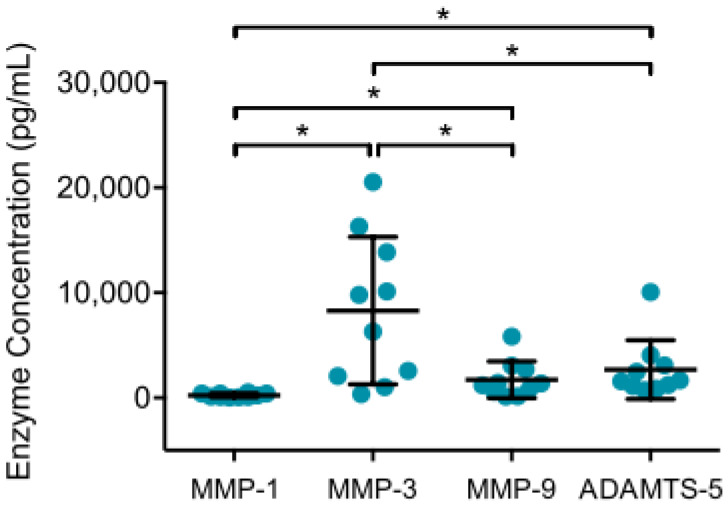
MMP and ADAMTS production in supernatant of synovial cell cultures are displayed. After enzymatic digestion of synovial membrane from patients with advanced knee OA, native synovial cells were cultured for 12 h at 37 °C and 5% CO_2_. Enzyme linked immunosorbent assays were then used to analyze enzyme production in culture supernatants. Mean and standard deviation of enzyme concentrations of MMP-1/-3/-9 and ADAMTS-5 are plotted (*n* = 12). Significant differences are indicated by asterisks: * *p* < 0.05. In addition, production of MMP-13, ADAMTS-5 and ADAMTS-7 were analyzed, but concentrations were below detection level.

To examine the inflammatory, chondrodestructive potential of synovial cells from patients with OA, ELISA was performed to analyze MMP and ADAMTS production in supernatants of native synovial cell cultures after 12 h. MMP-13 (*n* = 6), ADAMTS-4 (*n* = 8) and ADAMTS-7 (*n* = 8) concentrations were mostly below detection level, whereas considerable amounts of MMP-1, MMP-3, MMP-9 and ADAMTS-5 were detected. Mean MMP-3 values were significantly highest, followed by ADAMTS-5, MMP-9 and MMP-1, as shown in Figure 1. The detected MMP-9 and ADAMTS-5 concentration were significantly higher than MMP-1.

### 3.2. Chondrogenic MMP and ADAMTS Production

**Figure 2:**
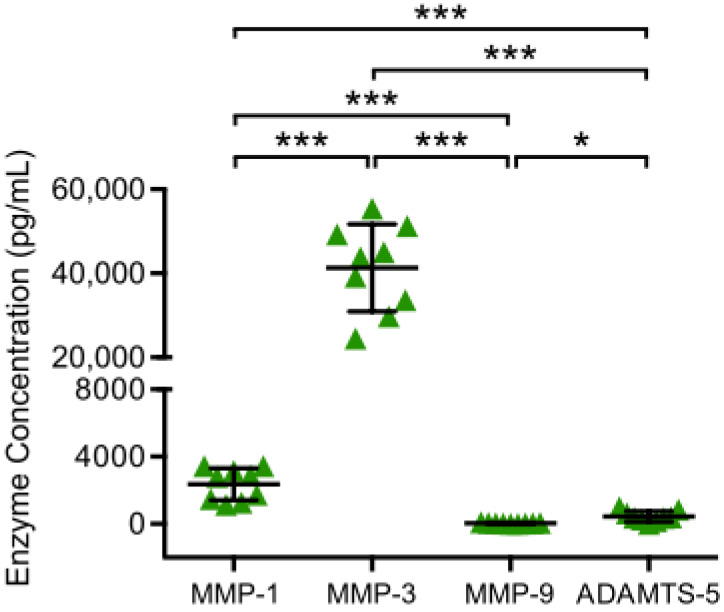
MMP and ADAMTS production of chondrocyte monocultures are displayed. Chondrocytes harvested from tibial plateau of patients with advanced knee OA were isolated and chondrocytes monocultures were incubated for 48 h at 37 °C. Then, enzyme concentrations in culture supernatants were analyzed performing enzyme linked immunosorbent assays. Mean and standard deviation of enzyme concentrations of MMP-1/-3/-9 and ADAMTS-5 are plotted (*n* = 9). Significant differences are indicated by asterisks: * *p* < 0.05; *** *p* < 0.001.

To determine catabolic chondrocyte activity, MMP and ADAMTS concentrations in supernatants of chondrocyte monocultures were analyzed by ELISA after 48 h of incubation (Figure 2). All analyzed enzymes (MMP-1, MMP-3, MMP-9 and ADAMTS-5) were detected. MMP-3 was the most highly enriched enzyme with a significantly higher concentration than MMP-1, MMP-9 and ADAMTS-5, while MMP-9 was detected with the lowest concentration, which was significantly lower than the concentration of MMP-1, MMP-3 and ADAMTS-5.

### 3.3. Impact of Synovial T Cell and Macrophage Depletion on MMP and ADAMTS Production

**Figure 3:**
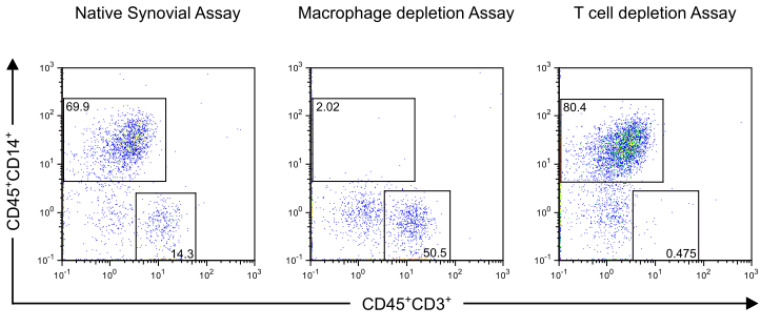
Flow cytometry analyses of synovial membrane from a representative patient with advanced knee OA before and after mononuclear cell depletion are shown. Synovial membrane was enzymatically digested and depletion of CD14^+^ and CD3^+^ cells were performed using magnetic activated cell sorting. Samples of native synovial assay and depletion assays were stained with R-phycoerythrin (PE)-conjugated antibodies against CD45 (Clone: 5B1), fluorescein isothiocyanate (FITC)-conjugated antibodies against CD14 (Clone: M5E2), Allophycocyanin (APC)-conjugated antibodies against CD3 (Clone: HIT3a) and 7-aminoactinomycin D (7-AAD). Alive cells were gated based on their forward-/side-scatter (FSC) profile and their affinity to 7-AAD. Dead cells and cell debris were excluded. Then mononuclear cells were defined by their FSC profile and their surface marker expression as CD45^+^CD14^+^ macrophages and CD45^+^CD3^+^ T cells. The effective depletion of synovial macrophages and T cells is shown.

Successful depletion was confirmed by flow cytometry (representative dot plot sample shown in Figure 3).

**Figure 4:**
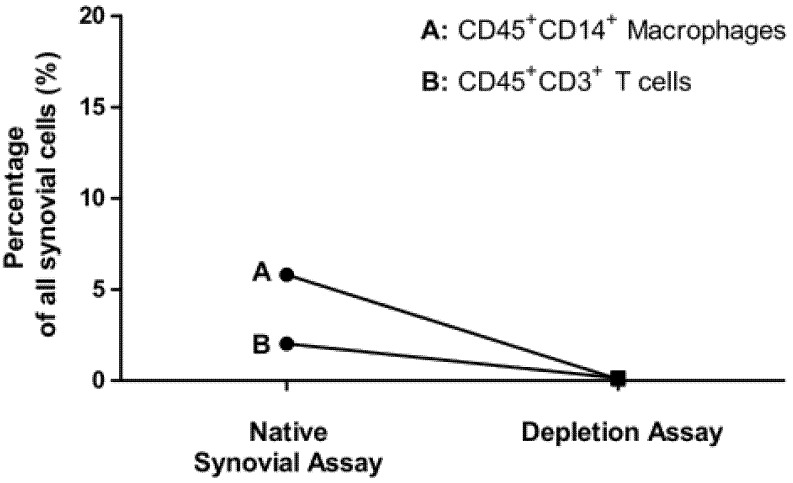
Depletion of synovial macrophages and T cells shown by flow cytometry analyses. Display of the mean values of: Percentage of CD45^+^CD14^+^ macrophages (**A**) and CD45^+^CD3^+^ T cells (**B**) on all living synovial cells in the samples of the native synovial assays and macrophages (*n* = 10) and T cell depletion assays (*n* = 12).

Percentage of CD45^+^CD14^+^ macrophages of all synovial cells was reduced from 7.63 ± 5.0% to 0.15 ± 0.05% (*n* = 10). Percentage of CD45^+^CD3^+^ T cells of all synovial cells was reduced from 2.02 ± 2.08 % to 0.14 ± 0.18 % (*n* = 12) as shown in Figure 4.

**Figure 5:**
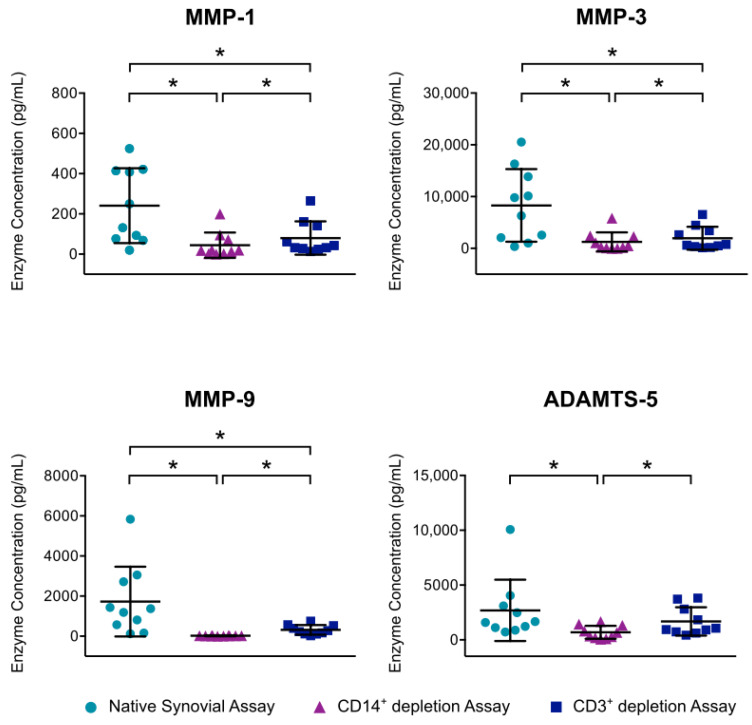
Effect of mononuclear cell depletion on MMP and ADAMTS production of synovial cell cultures is shown. After enzymatic digestion of synovial membrane from patients with advanced knee OA, depletion of CD14^+^ macrophages and CD3^+^ T cells was performed using magnetic activated cell sorting. Cells of native synovial assays and depletion assays were cultured for 12 h at 37 °C and 5% CO_2_. Enzyme linked immunosorbent assays were then used to analyze enzyme production in culture supernatants. Mean and standard deviation of enzyme concentrations of MMP-1/-3/-9 and ADAMTS-5 are plotted (*n* = 7). Significant differences are indicated by asterisks: * *p* < 0.05. In addition, production of MMP-13, ADAMTS-5 and ADAMTS-7 were analyzed, but concentrations were below detection level.

The mean MMP-1 concentration was significantly reduced from 241 pg/mL to 80 pg/mL by T cell depletion and to 63 pg/mL by macrophage depletion. Both macrophage and T cell depletion also resulted in a significant decline of MMP-3 values. Here, the mean concentration was reduced from 8290 pg/mL to 1953 pg/mL by T cell depletion and to 1259 pg/mL by macrophage depletion. Furthermore, MMP-9 concentration was significantly reduced by both macrophage depletion and T cell depletion (99%, resp. 82%). In addition, macrophage depletion resulted in a significant lessening of ADAMTS-5 compared to the concentration in native synovial cell culture. For all enzymes, macrophage depletion led to a significantly stronger reduction than T cell depletion (Figure 5).

### 3.4. Impact of CD4^+^CD127^dim/-^ Enriched PBMC on Catabolic Activity of Chondrocytes and the Effect of Treg

**Figure 6:**
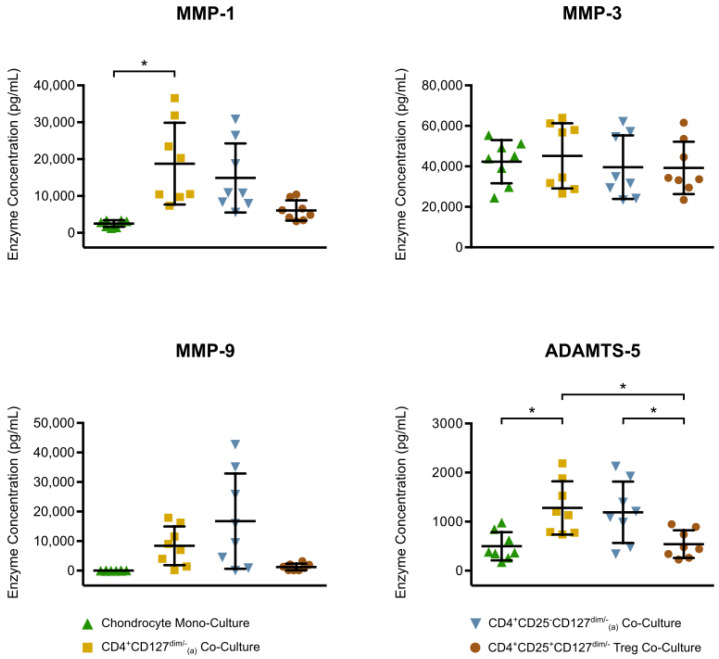
Quantitative analysis of MMP-1/-3/-9 and ADAMTS-5 concentration in chondrocyte monocultures and co-cultures with different CD4^+^ mononuclear cell subtypes is displayed. Chondrocytes harvested from tibial plateau of patients with advanced knee OA were isolated. A population of CD4^+^CD127^dim/-^-enriched PBMC and two subpopulations consisting of CD4^+^CD25^-^CD127^dim/-^-enriched PBMC (Treg-depleted enriched PBMC) and CD4^+^CD25^+^CD127^dim/-^ Treg were isolated from heparin anti-coagulate peripheral blood using Ficoll-PlaqueTM and magnetic activated cell sorting. Chondrocytes monocultures and co-cultures were incubated for 48 h at 37 °C and then enzyme concentrations in culture supernatants were analyzed performing enzyme-linked immunosorbent assays. The mean and standard deviation of enzyme concentrations of MMP-1/-3/-9 and ADAMTS-5 are plotted (*n* = 8). Significant differences are indicated by asterisks: * *p* < 0.05.

In order to investigate the interaction between mononuclear cells and chondrocytes, a series of co-culture experiments were set up and the enzyme release was assessed (Figure 6). Compared to chondrocyte monoculture, a significant increase of more than six-fold was detected for MMP-1 in chondrocyte co-culture with CD4^+^CD127^dim/-^-enriched PBMC. The mean ADAMTS-5 concentration, which was 498 pg/mL in the chondrocyte monoculture, was also significantly increased to 1279 pg/mL in the chondrocyte co-culture with CD4^+^CD127^dim/-^-enriched PBMC. The enzyme concentrations between the chondrocyte monoculture and the two chondrocyte co-cultures with Treg and Treg-depleted, CD4^+^CD25^-^CD127^dim/-^-enriched PBMC showed no significant differences. However, all enzymes were detected in the Treg co-culture at lower concentrations compared to the other co-cultures, which was significant for ADAMTS-5. Furthermore, no significant differences in enzyme concentrations were shown between co-culture with CD4^+^CD127^dim/-^-enriched PBMC (including Treg) and co-culture with Treg-depleted, CD4^+^CD25^-^CD127^dim/-^ enriched-PBMC.

## 4. Discussion

There is substantial evidence that metalloproteinases (MMP and ADAMTS), which are capable of degrading collagen and aggrecan as the main extracellular components of hyaline cartilage, are significantly involved in OA pathology [11,12,13,14,15]. However, it is still largely unknown which cell types and enzymes are mainly responsible for the catabolic and enzymatic turnover in OA disease. In particular, the role of T cells and whether they amplify the release of cartilage-degrading enzymes and are thus actively involved in OA progression is yet unknown.

The overarching goal of this study was to analyze the impact of synovial cells and especially synovial macrophages and T cells on the release of MMP (MMP-1,-3,-9, and -13) and ADAMTS (ADAMTS-4,-5, and -7) in order to determine their inflammatory chondrodestructive potential in OA pathology.

While previous studies suggest that MMP-13 and ADAMTS-4/-7 are involved in OA pathology [14,15,16], concentrations of these enzymes in supernatants of synovial cell cultures were mostly below detection level in our study. Our results suggest that the synovial membrane is not a major source of these enzymes, at least in patients with advanced knee OA. MMP-13 is already known as a metalloproteinase, which is preferentially expressed by chondrocytes [36]. In contrast to our findings a previous study, which also included patients with hip OA, detected MMP-13 in supernatants of synovial cell cultures and detected the synovial expression of ADAMTS-4 [19]. Another study analyzing OA SF revealed the highest ADAMTS-4 concentration in early disease stages [37]. Thus, MMP-13 and ADAMTS-4 might present different secretion profiles depending on OA location and stage.

The results of our study show that the synovial cells of patients with advanced knee OA produce mainly collagenase MMP-1, stromelysin MMP-3, gelatinase MMP-9 and aggrecanase ADAMTS-5, which is in accordance by a previous report in which these enzymes were detected in the supernatants of synovial cell cultures by ELISA or their synovial expression by PCR [19].

In our study, MMP-3 was found to possess the highest enzyme concentration in comparison to MMP-1, MMP-9 and ADAMTS-5, both in supernatants of synovial cell cultures and in supernatants of chondrocyte monocultures. In line with our findings, previous studies have also shown that expression of MMP-3 is significantly increased in OA synovial tissue and peripheral blood [12,38]. These results suggest that MMP-3, which is capable of degrading non-collagenous matrix proteins [39] may play a central role in the cartilage degradation process.

The fact that all detected enzymes (MMP-1, MMP-3, MMP-9, ADAMTS-5) belong to different subtypes of metalloproteinases, which are capable of degrading various extracellular components of cartilage, underlines the chondrodestructive potential of synovial cells in OA.

As a next step, we investigated whether synovial T cells and macrophages were involved in this metalloproteinase release. T cell prevalence in OA SM has been confirmed by several studies [24,25,27,28,40,41]. Synovial T cells of OA patients were shown to present an activated state [41] and T cell infiltration correlates with different OA subtypes [28]. Whether T cells are involved in the release of chondrodestructive metalloproteinases in OA joints and thus actively affect the cartilage degradation process has not been known so far. To our knowledge, this is the first study in which the depletion of synovial T cells from patients with OA has been successfully performed. The T cell depletion led to a significant decline of MMP-1, MMP-3 and MMP-9 concentration in synovial cell culture supernatants indicating that synovial T cells might actively contribute to the cartilage degradation process by inducing the release of chondrodestructive metalloproteinases.

Macrophage depletion was performed utilizing MACS CD14 depletion assay and confirmed by flow cytometry. CD14 is a marker most commonly used for macrophage characterization in the field of OA research, mostly as sole marker [42,43,44,45,46]. Other markers, such as CD68, showed high expression in human fibroblasts, which is one of the major cellular components of synovial tissue [47,48], whereas expression of CD14 is very specific for macrophages in synovial tissue samples. CD14^+^ macrophage depletion led to a significantly stronger reduction of MMP-1,-3,-9 and ADAMTS-5 compared to T cell depletion, suggesting that macrophages have a greater impact on the release of these enzymes. Previous studies already suggested that macrophages play a central role in OA pathology and disease progression [19,20,49]. In this study, depletion of synovial macrophages resulted in a significant reduction of MMP-1 (82%), MMP-3 (85%), MMP-9 (99%) and ADAMTS-5 (74%) concentration in culture supernatants, underlining that macrophages are significantly involved in the release of these metalloproteinases and thus participate actively in enzymatic cartilage degradation process in OA pathology.

Interestingly, both macrophage depletion and T cell depletion resulted in a strong diminishing of MMP-1 (82%, resp. 67%), MMP-3 (85%, resp. 76%) and MMP-9 (99%, resp. 82%) concentration. These results suggest that macrophages and T cells do not induce the secretion of these enzymes independently, but by interacting with other cells such as fibroblasts or with each other. This interaction could be exerted by direct cell contact [50] or through paracrine processes [51]. It has already been suggested that there might be a synovial macrophage-fibroblast interaction leading to increased levels of metalloproteinases in an OA joint [19]. Further studies to investigate whether there are T cell-macrophage–fibroblast interactions in the synovial membrane leading to an increased enzymatic burden in OA joints are still to be performed.

Since it is considered that chondrocytes themselves are involved in the cartilage degradation process, we also investigated the metabolic activity through MMP, ADAMTS production of chondrocytes from patients with advanced knee OA. All analyzed metalloproteinases (MMP-1, MMP-3, MMP-9, ADAMTS-5) were detected in supernatants of chondrocyte monocultures. These results support the hypothesis that the chondrocytes themselves contribute to enzymatic burden in OA joints and thus to disease progression [17]. Since this study does not include monocultures of chondrocytes from healthy donors due to ethical restrictions, we cannot prove that MMP values were certainly pathological in our study.

Based on previous studies showing that CD4^+^ T cells are present in SF of OA patients [27,29], indicating that these cells may interact with chondrocytes in vivo, we also investigated the impact of different PBMC subtypes including Treg on MMP and ADAMTS production in a chondrocyte co-culture experiment. Knowledge about the interaction of inflammatory cells with chondrocytes, which might lead to a catabolic milieu in OA joints, is still limited. It is also unknown whether Treg have a suppressive effect on the catabolic chondrocyte activity or other inflammatory cells in OA joints. In this study, chondrocyte co-culture with CD4^+^CD127^dim/-^-enriched PBMC resulted in a significant increase of MMP-1 and ADAMTS-5 concentration compared to chondrocyte monoculture. These results suggest that CD4^+^CD127^dim/-^ cells in SF are able to actively contribute to the enzymatic burden in OA joints, most likely by direct cell contact, as described before [52]. However, the mechanisms leading to the increased metalloproteinase level in chondrocyte co-culture with CD4^+^CD127^dim/-^-enriched PBMC compared to chondrocyte monoculture were not analyzed in this study. A comparison of enzyme concentration in supernatants of chondrocyte co-cultures with CD4^+^-enriched PBMC with or without Treg revealed no significant differences. Furthermore, no significant differences were found between chondrocyte monocultures and co-cultures with Treg concerning MMP and ADAMTS concentration. Therefore, a suppressive effect of Treg on metalloproteinase release cannot be demonstrated in this study. Our results only allow the conclusion that Treg, in the presence of chondrocytes, have the least effect on metalloproteinase production compared to the other PBMC subtypes studied. Further studies are needed to analyze the role of Treg and especially the effect of Treg on effector T cells in OA.

## 5. Conclusions

The results of this study support the hypothesis that OA is not a mere degenerative disease, but a multifactorial disease including inflammatory processes in different articular tissues such as synovial membrane and cartilage, eventually leading to OA progression. To the best of our knowledge, this is the first study demonstrating that not only synovial macrophages but also synovial T cells from patients with advanced OA have a catabolic potential by inducing the release of chondrodestructive metalloproteinases—especially MMP-1, MMP-3 and MMP-9. In addition, this study supports the hypothesis that mononuclear cells contribute to an increased catabolic milieu in the presence of chondrocytes, which may eventually result in cartilage degradation and thus OA progression.

A better understanding of the inflammatory processes in OA—in particular, of the interaction between macrophages, T cells and fibroblasts—has a great potential to discover new treatment pathways that have not only symptomatic but also causal effects. Treating OA patients causally—especially those with early OA—could be a major breakthrough for individuals and health care systems worldwide due to the high prevalence and enormous physical and social limitations of the disease. OA patients could be prevented from suffering chronic pain with poor joint function and quality of life for many years.

Our results suggest that therapeutic strategies aimed at inhibiting the inflammatory processes in OA to halt disease progression should not only target macrophages, but also T cells.

## Figures and Tables

**Table 1:** Study population.

	Total Study Population
Number of patients, *n*	21
Gender, *n* (%)	
Male	4 (19%)
Female	17 (81%)
Age, years	66.0 ± 12.5
BMI (kg/m^2^)	28.0 ± 4.9
K&L score, *n* (%)	
3	8 (38.1%)
4	13 (61.9%)
Operation side *n* (%)	
Right	13 (61.9%)
Left	8 (38.1%)
Operation type	
UKA, *n* (%)	10 (47.6%)
TKA, *n* (%)	11 (52.4%)
Leucocytes/nL	7.0 ± 2.0
C-reactive protein (mg/L)	3.0 ± 1.7

Sociodemographic and clinical parameters of the study population are displayed. Data are presented as mean ± standard deviation (SD). BMI = body mass index; K&L score = Kellgren and Lawrence score; UKA = unicompartmental knee arthroplasty; TKA = total knee arthroplasty.

## Supplementary Materials

The following are available online at https://www.mdpi.com/2077-0383/9/5/1279/s1, Figure S1: Treg isolation.
