# Supplementary material for: Impact of Mononuclear Cell Infiltration on Chondrodestructive MMP/ADAMTS Production in Osteoarthritic Knee Joints—An Ex Vivo Study
Source: J Clin Med. 2020 Apr 28;9(5):1279. doi: 10.3390/jcm9051279 (PMC7288002; doi:10.3390/jcm9051279)
Supplement: Supplementary file 1 [file jcm-09-01279-s001.pdf]

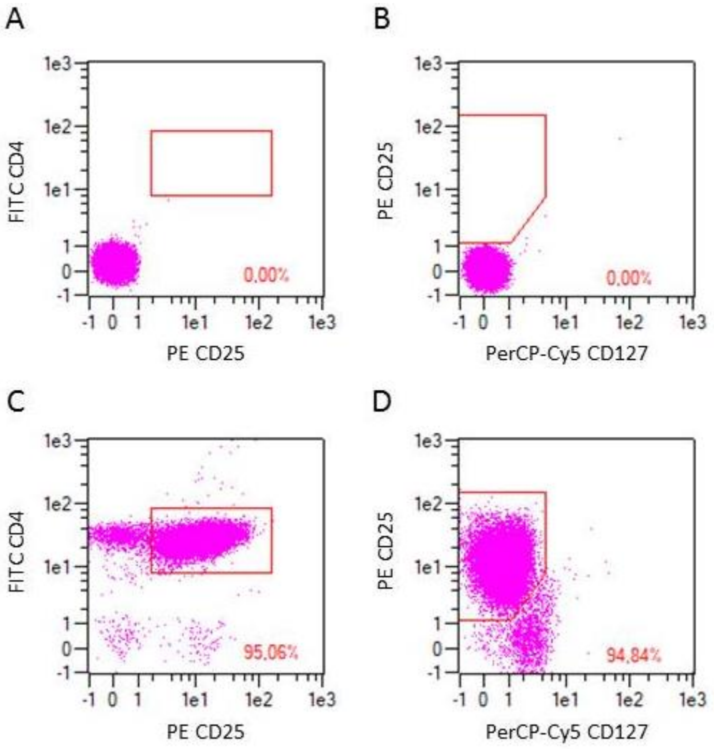

Supplementary Figure S1:

In the study presented the inflammatory impact of $CD4^{+}CD127^{dim/-}$ enriched PBMC, $CD4^{+}CD25^{+}CD127^{dim/-}$ regulatory T cells (Treg) and a population of Treg depleted $CD4^{+}CD25^{-}CD127^{dim/-}$ enriched PBMC was analyzed in a chondrocyte co-culture experiment. In preliminary studies we confirmed successful Treg isolation process using the $CD4^{+}CD25^{+}CD127^{dim/-}$ Regulatory T Cell Isolation Kit II human (Miltenyi Biotec, Germany) by flow cytometry.

A representative dot plot confirming successful Treg isolation (~95%) is shown here.

A/B: Autofluorescence, C: FITC CD4 PE CD25 staining, D: PE CD25 PerCP-Cy5 CD127 staining
